# Postmarketing safety of orphan drugs: a longitudinal analysis of the US Food and Drug Administration database between 1999 and 2018

**DOI:** 10.1186/s13023-021-02166-9

**Published:** 2022-01-04

**Authors:** Min Fan, Adrienne Y. L. Chan, Vincent K. C. Yan, Xinning Tong, Lauren K. W. Lau, Eric Y. F. Wan, Eliza Y. T. Tam, Patrick Ip, Terry Y. Lum, Ian C. K. Wong, X. Li

**Affiliations:** 1 Centre for Safe Medication Practice and Research, Department of Pharmacology and Pharmacy, Li Ka Shing Faculty of Medicine, The University of Hong Kong, L2-59, 2/F, Laboratory Block, Faculty of Medicine Building, 21 Sassoon Road, Pokfulam, Hong Kong; 2 Laboratory of Data Discovery for Health (D24H), Hong Kong Science Park, Sha Tin, Hong Kong; 3 Groningen Research Institute of Pharmacy, Unit of PharmacoTherapy, -Epidemiology and -Economics, University of Groningen, Groningen, The Netherlands; 4 Department of Medicine, Li Ka Shing Faculty of Medicine, The University of Hong Kong, Pokfulam, Hong Kong; 5 Department of Family Medicine and Primary Care, Li Ka Shing Faculty of Medicine, The University of Hong Kong, Pokfulam, Hong Kong; 6 Department of Paediatrics and Adolescent Medicine, Li Ka Shing Faculty of Medicine, The University of Hong Kong, Pokfulam, Hong Kong; 7 Department of Social Work and Social Administration, Faculty of Social Sciences, The University of Hong Kong, Pokfulam, Hong Kong; 8 Research Department of Practice and Policy, UCL School of Pharmacy, London, UK

**Keywords:** Orphan drug designation, Adverse events, Postmarketing surveillance, Medication safety, Risk–benefit trade-off

## Abstract

**Background:**

Information about the specific regulatory environment of orphan drugs is scarce and inconsistent. Uncertainties surrounding the postmarketing long-term safety of orphan drugs remain. This study aimed to evaluate the labelling changes of orphan drugs and to identify postmarketing safety-associated approval factors.

**Methods:**

This retrospective cohort study includes all drugs with orphan drug designation approved by the Center for Drug Evaluation and Research of the US Food and Drug Administration between 1999 and 2018. Main outcomes are safety-related labelling changes up to 31 December 2019. We defined any safety-related labelling changes as postmarketing safety events (PMSE). Safety-related withdrawals, suspensions, and boxed warnings were further categorised as severe postmarketing safety events (SPSE). Outcome measurements include frequencies of PMSE, SPSE, and association between approval factors and the occurrence of safety events.

**Results:**

Amongst the 214 drugs identified with orphan drug designation (25.7% biologics), 83.6% were approved through at least one expedited programme, and 29.4% were approved with boxed warnings. During a median follow-up of 6.74 years since approval, 69.2% and 14.5% of the analysed orphan drugs had PMSE and SPSE, respectively. Safety-related withdrawal (0%, 0/214), suspended marketing (0.46%, 1/214) and new boxed warnings are uncommon (3.7%, 8/214). The safety-related labelling changes were more frequent in the drugs approved with boxed warnings [Incidence rate ratio (IRR): 1.95 (1.02–3.73)] and approved for long-term use [IRR: 2.76 (1.52–5.00)].

**Conclusions and Relevance:**

In this long-term postmarketing analysis, approximately 70% of FDA-approved orphan drugs had safety-related labelling changes although severe safety events were rare. While maintaining early access to orphan drugs, the drug regulatory body has taken timely regulatory action with postmarketing surveillance to ensure patient safety.

**Supplementary Information:**

The online version contains supplementary material available at 10.1186/s13023-021-02166-9.

## Introduction

In the United States (US), orphan drug designation refers to a special status granted by the Food and Drug Administration (FDA) to drugs that are indicated for a disease that affects 200,000 or fewer persons in the US or drugs with no reasonable expectation that sales will offset the costs of development and marketing [1]. Sponsors may apply for orphan drug designation at any point during the drug development process before submitting the marketing authorisation application. Drugs with orphan designation may be subject to research, development, approval and regulatory benefits such as tax credits for qualified research expenses, and waiver of the Prescription Drug User Fee [2]. To accelerate patients’ access to orphan drugs, some orphan drugs may be approved via one of four FDA expedited programmes, namely, priority review, breakthrough therapy, fast-track designations, and accelerated approval pathway [3]. Under certain circumstances, Phase II safety trials may be used as pivotal trials; similarly, Phase II and III trials may be combined when the patient population is exceptionally low that large trials are not logistically feasible [4].

Since the enactment of the Orphan Drug Act in 1983, newly approved chemical agents and biologics with orphan drug designation rose from 12.7% during 1995–1997 to 38.1% in 2015–2017 of all FDA-approved drugs [5]. Meanwhile, the long-term safety of orphan drugs is still uncertain, partially due to short follow-up duration, small sample sized- or single armed- clinical trials and the accelerated approval process [6]. To ensure patients’ access to life-saving treatment, it is not societally desirable to keep a drug at the testing phase until all possible safety considerations are determined. Postmarketing surveillance with regulatory action provides complementary evidence to the safety reports at trial stage to enhance patient safety. However, most postmarketing safety studies of orphan drugs are focused on individual drugs [7, 8]. As such, evidence of orphan drug safety collectively remains scarce and inconsistent while heterogeneity across studies renders the synthesis of results infeasible. Other orphan drug safety studies may have included all novel therapeutics with limited insight on the regulatory environment of orphan drugs. This presents challenges for patients with rare diseases and clinicians in understanding the process behind orphan drug approval, many of whom may already be deterred by the inherent uncertainty of disease progression, and thus overestimate the risks alongside newly approved orphan drugs.

As the demand for treating rare diseases is immense and highly time-sensitive, the postmarketing safety of orphan drugs and the drug approval environment should be judiciously evaluated to inform treatment access and monitoring decisions. By extracting longitudinal data from the FDA orphan drug database, this study aimed to 1) describe the landscape of long-term postmarketing safety of FDA approved orphan drugs; and 2) assess the association between approval factors and the occurrence of postmarketing safety events to ensure that clinicians and patients have the appropriate information to evaluate the risks and benefits for their particular rare condition.

## Methods

### Orphan drug identification

We analysed all drugs with orphan drug designation approved by the FDA between 1999 and 2018 [9]. The new drug list was extracted from the *New Molecular Entity and Original Biologic Approvals Annual Reports* provided by the Center for Drug Evaluation and Research (CDER), FDA (hereinafter referred to as Reports) [10, 11]. The *Compilation of CDER dataset* was used to reconfirm the data from Reports [12]. The approval date and orphan drug designation status were then verified using the FDA’s *Orphan Drug Product Designation Database *[13].

### Approval characteristics

Information on approved orphan drugs were extracted from drug labels, approval letters, approval reviews, and other approval documents uploaded onto *Drugs@FDA*. Extracted drug information included brand name, generic name, manufacturer, orphan drug designation date, approval date, approval status, product type [New Molecular Entity Application (NME) or New Biologic License Application (BLA)], therapeutic area, expedited programmes, approved with boxed warning, and approved for long-term use. Categories of approval information were determined based on a previous study [14]. Additional files 1 and 2 summarises data collection flow and details the data extraction variables.

The therapeutic category of each analysed drug was based on the corresponding Anatomical Therapeutic Classification (ATC, third level) according to the World Health Organization ATC Index 2020 [15]. For drugs with an ATC code, the authors MF and AYLC independently categorised these into the respective therapeutic areas based on the active ingredients and indications from the drug labels. Any discrepancies in categorisation were further confirmed by author VKCY, a registered pharmacist.

The FDA has four expedited approvals programmes (Table 1). In this study, we analysed orphan drugs as those approved through expedited programmes with ‘priority review’, ‘breakthrough therapy’ or ‘accelerated approval’ recorded in the drug approval documents. The fast-track designation, implemented only since 2004, was not assessed in the current analysis. ‘Approved with boxed warning’ was defined as the presence of a boxed warning on the initially approved label on *Drugs@FDA*. ‘Approved for long-term use’ was defined as chronic or repeat intermittent use for 6 months or longer, based on information in the ‘Indication and Usage’ and ‘Dosing and Administration’ sections of the initially approved label, or in information regarding the length of treatment found on the label. Keywords such as ‘cancer,’ ‘chronic,’ ‘long term use,’ and ‘repeated’ on the drug label were also used to categorise duration of use.

**Table 1:** Comparison of the four FDA expedited programmes

Expedited programme	Type	Effect
Priority review	Designation	Reduces time of application review process from 10 to 6 months of priority regulatory review
Breakthrough therapy	Designation	Expedites review of drugs that may show substantial improvement for patients with serious diseases over existing drugs
Fast track	Designation	Expedites drug development and review to treat serious conditions and fill unmet medical need
Accelerated approval	Approval pathway	Permits use of surrogate or intermediate clinical endpoint for filling an unmet medical need for serious conditions

### Outcomes

Following the product launch, updated safety events reported by manufacturers, health professionals, and consumers are continuously collected by the FDA via MedWatch: The FDA Safety Information and Adverse Event Reporting Program [16]. These reports are made available on the FDA Adverse Event Reporting System for assessment by clinical reviewers [16]. Depending on the evidence presented and the severity of reported adverse events, these may be developed into product labels to inform prescription practice. The primary outcome of the study is postmarketing safety events (PMSE) resulting in labelling changes. We defined PMSE as any safety-related label change after approval including boxed warnings, contraindications, warnings and precaution, adverse reactions, drug interactions, withdrawal, and suspended marketing. The secondary study outcome is severe postmarketing safety events (SPSE), a subgroup of PMSE that considered safety-related withdrawals, suspensions, and boxed warnings post-approval. Safety-related label changes from the date of drug approval to 31 December 2019 were extracted from *FDA MedWatch* and *Drugs@FDA*. For drugs with multiple approved indications, the follow-up began from the first approval date with orphan drug designation to the study end date.

### Statistical analysis

Descriptive statistics were used to summarise the characteristics of included orphan drugs. Mean ± standard deviation (SD), median ± interquartile range (IQR), and frequencies with percentages were reported as appropriate. Negative binomial regression was applied to assess the association between approval factors and the cumulative number of PMSE within 5-years after approval. Maximum observational time, from approval to 5 years or to the study end date, was included as an offset variable in the regression. Kaplan–Meier estimates with log-rank tests were applied to compare the risk of SPSE over time amongst the following binary variables: (1) product type (NME versus BLA); (2) therapeutic area (antineoplastic versus non-neoplastic; (3) priority review; (4) accelerated approval; (5) breakthrough therapy; (6) approved for long-term use; (7) approved with boxed warning. Multivariable Cox regression was applied to assess the association between the seven factors mentioned above and the occurrence of SPSE starting from the respective drug approval date to the date of first SPSE or 31 December 2019, whichever was earlier. A two-sided *P* value of less than 0.05 was considered statistically significant. Schoenfeld residual-based test was used for testing the proportional hazard assumption. R version 3.6.0 (R Foundation for Statistical Computing, Vienna, Austria) was used for data manipulation and analysis. The programming and results were cross-checked for consistency by MF and AYLC.

## Results

### Characteristics of FDA-approved orphan drugs

We identified 214 drugs with orphan drug designation approved by the US FDA between 1 January 1999 and 31 December 2018. Amongst these, 25.7% (n = 55) were biologics, and the remaining were small molecule drugs (Table 2). The most common therapeutic areas were antineoplastic and immunomodulating agents (46.3%), followed by alimentary tract and metabolism (12.1%), and nervous system (7.5%). Around four-fifths (83.6%) of the reviewed drugs were approved via at least one of the considered expedited programmes. Over a quarter (29.4%) were approved with boxed warnings. Around twelve percent (n = 27) of the analysed orphan drugs had multiple indications, all of which had orphan drug designation at first approval.

**Table 2:** Characteristics of orphan drugs approved by the FDA from 1999 to 2018

Characteristics	Number (%)
Novel orphan drugs	214
Follow-up years since approval [median (IQR)]	6.7 (3.0–12.6)
*Therapeutic area*	
Antineoplastic and immunomodulating agents	99 (46.3)
Alimentary tract and metabolism	26 (12.1)
Nervous system	16 (7.5)
Blood and blood forming organs	13 (6.1)
Various	13 (6.1)
Cardiovascular system	9 (4.2)
Systemic hormonal preparations, excl. sex hormones	9 (4.2)
Anti-infective for systemic use	8 (3.7)
Musculo-skeletal system	8 (3.7)
Antiparasitic products, insecticides and repellents	6 (2.8)
Respiratory system	5 (2.3)
Sensory organs	1 (0.5)
Therapeutic radiopharmaceuticals	1 (0.5)
*Approval status*	
Priority review	169 (79.0)
Accelerated approvals	55 (25.7)
Breakthrough therapy	53 (24.8)
Approved with boxed warning	63 (29.4)
For long-term use	73 (34.1)
*All postmarketing safety events up to 31 December 2019*	
Number of safety events	641
Withdrawal	0 (0)
Suspended marketing	1 (0.16)
Boxed warning	48 (7.49)
Contraindications	50 (7.80)
Drug interactions	68 (10.61)
Warnings and precautions	453 (70.67)
Adverse reactions	443 (69.11)

### Postmarketing safety events

**Fig. 1:**
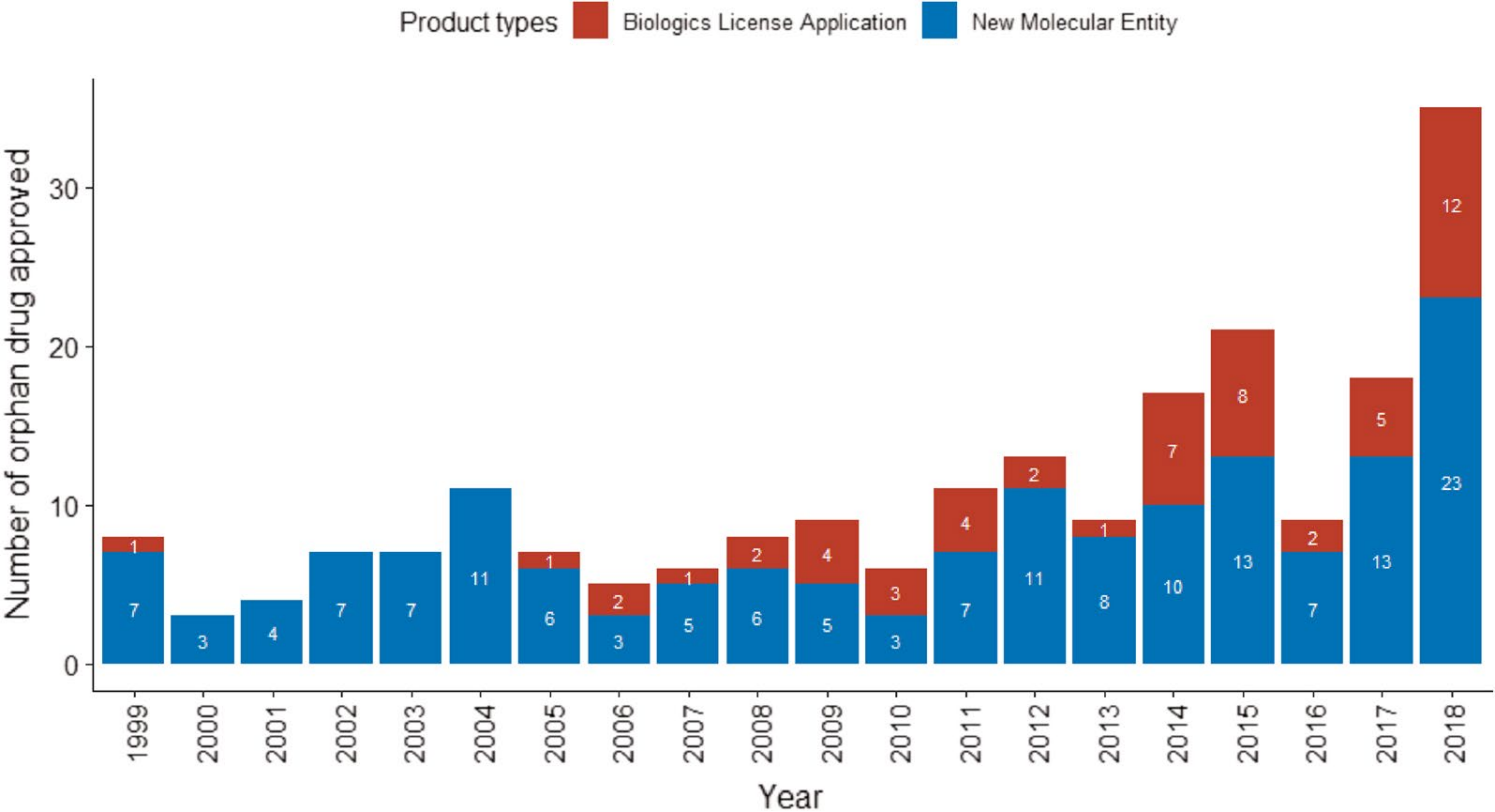
Timeline of orphan drugs approval and safety-related label changes, by product type

The number of FDA approved orphan drugs in general increased between 1999 and 2018. Figure 1 illustrates the timeline for orphan drugs approval and safety-related labelling changes. Of the approved orphan drugs, 69.2% (n = 148) were affected by at least one PMSE during a median follow-up time of 6.74 years. In total, there were 641 labelling changes related to postmarketing safety (boxed warning: 48; suspended marketing: 1; contraindications: 50; drug interactions: 68; warnings and precautions: 453; adverse reactions: 443, one label update could include multiple safety events). Of the analysed drugs 14.5% (n = 31) had SPSE with 49 labelling changes (safety-related withdrawals: 0; suspended marketing: 1; and boxed warnings: 48). The average time to first SPSE was 4.0 (SD: 3.9) years. New boxed warnings were added to eight drugs (Additional file 3) while the remaining 23 had reinforcements to the initial boxed warnings. Only one drug, Iclusig (ponatinib), had a temporary marketing suspension in 2013 because of the risk of life-threatening blood clots and severe narrowing of blood vessels. However, given the narrow population group, the benefits were considered to outweigh the risks and it was replaced on the market after two months.

### Approval factors and the occurrence of safety events

**Fig. 2:**
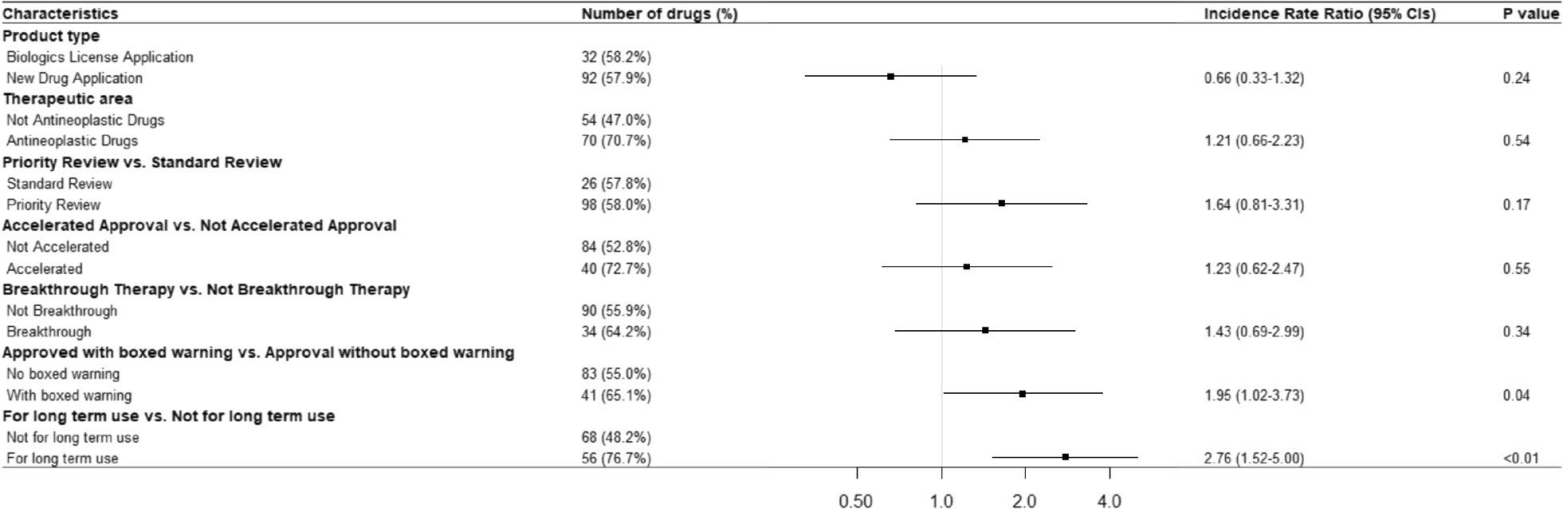
Association between drug approval factors and postmarketing safety events within 5 years of approval. Negative binomial regression was applied to assess the associations between approval factors and the cumulative number of PMSE within 5-years after approval. *CI* confidence interval, *PMSE* postmarketing safety events, *yrs* years

**Fig. 3:**
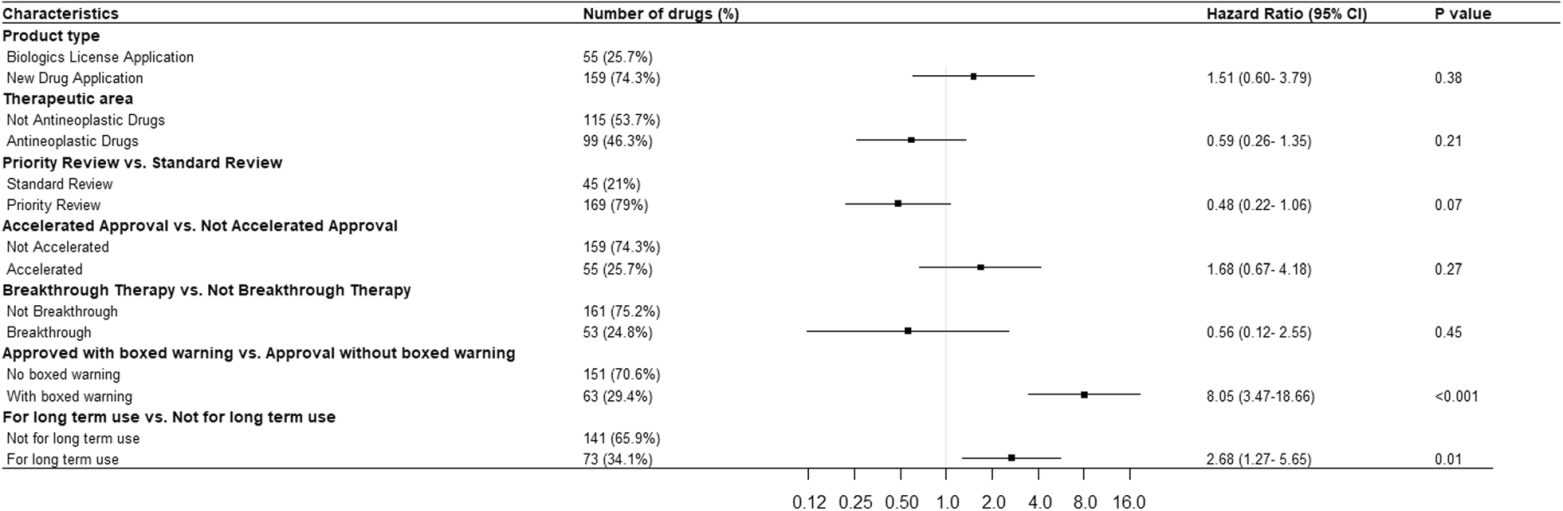
Association between drug approval status and severe postmarketing safety events. Cox Proportional-Hazards regression model was applied to assess the association between multiple approval factors and the time-to-SPSE

Negative binomial regression show that ‘approved for long-term use’ [Incidence rate ratio (IRR): 2.76, 95% confidence interval 1.52–5.00] and ‘approved with boxed warning’ (IRR: 1.95, 95% CI 1.02–3.73) are two independent approval factors significantly associated with the frequency of PMSEs within 5 years since approval (Fig. 2). In the log-rank test, we observed a significantly increased proportion of SPSE among drugs with priority review during approval (*p* = 0.01), approved with boxed warning (*p* < 0.001), and approved for long-term use (*p* = 0.04) than those without (Additional file 4). We conducted a multivariable Cox proportional-hazards regression with all approval factors included. The Schoenfeld residuals test showed that the proportional hazard assumption was met for all the included variables and no significant violations were observed (Additional file 5). The Cox model confirmed similar findings that drugs ‘approved for long-term use' [Hazard ratio (HR): 2.68, 95% CI 1.27–5.65] and ‘approved with boxed warning’ (HR: 8.05, 95% CI 3.47–18.66) were independently significantly associated with the occurrence of SPSE (Fig. 3).

## Discussion

### Summary of findings

This study provides an overview of orphan drug safety through the first, most comprehensive longitudinal analysis of the FDA database on orphan drugs. Our analysis includes all FDA-approved orphan drugs since 1998 with up to 20 years of postmarketing surveillance. Of the 214 FDA-approved orphan drugs, 69.2% had labelling changes related to PMSE since designation and approval. In combination with available evidence from the drug regulatory agency and academic literature, this study reconfirms that timely regulatory action has been in place for orphan drugs with frequent safety-related labelling updates to inform prescription practice.

In our study, one of the approval factors associated with the frequency of PMSEs at 5-years after approval is ‘approved for long-term use’. This suggests a potential cumulative dose effect from the long-term use of a drug. However, this result could potentially be prone to survival bias, given that patient factors and disease trajectories commonly associated with chronic rare diseases differ from more rapidly progressive rare diseases. Patients with rapidly progressive rare diseases may not survive long enough to experience PMSE induced by the drug, and thus less safety reports may be generated. As such, when given sufficient sample sizes, longitudinal studies using electronic medical records may provide further insight regarding the safety of orphan drugs for short- and long-term use, where patient factors can be taken into consideration.

When studying orphan drugs safety, SPSE is a more pertinent consideration than PMSE due to the limited or even absent treatment options for life-threatening rare safety events. In our study, over 15% of FDA-approved orphan drugs had labelling changes related to SPSE, mostly as the reinforcement of initial boxed warnings issued on approval. Newly added boxed warnings only accounted for a small proportion overall. Drugs approved with boxed warnings had earlier labelling updates related to SPSE in multivariable analysis. This is further indication of enhanced label updates for drugs approved with boxed warnings, the evident interaction between pre- and postmarketing regulation and the importance of long-term safety surveillance.

### Comparison with other studies

As mentioned previously, few studies had focused on postmarketing safety specific to orphan drugs. Onakpoya et al. assessed the safety of 74 orphan drugs approved by the European Medicines Agency (EMA) between 2002 and 2014 [17]. The study reported that 86.5% of identified orphan drugs had evidence of serious adverse events, a much higher proportion than our study. As with our study, orphan drugs approved for treating cancerous conditions had a higher proportion of adverse events. However, the definition of adverse events was unclear, and the study employed academic databases for evidence regarding orphan drug safety, where reporting and publication biases may exist. Meanwhile, the association between regulatory approval factors and postmarketing safety remain unexplored.

In a broader literature review, several studies that evaluated postmarketing drug safety potentially included a subgroup of orphan drugs, such as new molecular entities, new therapeutic biologics, and drugs that lack safety and efficacy data [14, 18–28]. One study on postmarketing safety of FDA-approved novel therapeutics showed that orphan status was not significantly associated with PMSE [14]. Studies using FDA and EMA databases found that novel therapeutics or biologics with accelerated approval or shorter time to obtain approval, respectively, experienced a higher rate of PMSE [14, 18, 19]. This finding was consistent with our multivariable analysis which focused only/?solely on orphan drugs. Furthermore, these studies focused predominantly on PMSE rather than SPSE.

Caution must be exercised when contextualising these findings from non-orphan drug specific studies as drugs with orphan drug designation might experience different review, surveillance and reporting procedures. Moreover, approval factors are not necessarily comparable among different drug approval agencies. Orphan drugs with identified risk factors for SPSE, namely ‘for long-term use’ and ‘approved with boxed warnings’, should be further examined using real-world data and multiple drug regulatory databases to inform safety monitoring processes.

### Implications and future research directions

Findings from this study will inform multiple stakeholders about the frequency of safety-related labelling changes in orphan drugs detected by the FDA. This reinforces the role of postmarketing safety surveillance—to allow health professionals to be updated on any safety-related events for new orphan drugs alongside the predominant benefits to patients with rare diseases. Despite poor prognoses and limited treatment options, patients with rare diseases may be open towards drugs with more uncertainties than traditionally accepted. Decision-makers are therefore challenged to make trade-offs between conclusive safety evidence and timely life-saving treatment to address unmet patient needs. Quality safety data from structured surveillance programmes will assist regulators and payers to better mitigate uncertainties and balance the risks and benefits without further exposing patients to treatments with unproven benefits. Establishing orphan drugs or rare disease registries is imperative for extensive and continuous safety (and effectiveness) monitoring.

Future research should consider aggregating data from various drug surveillance databases to achieve power for more nuanced orphan drug safety assessment. Suggested databases include the safety and approval databases from the EMA, and drugs approved by the Center for Biologics Evaluation and Research of FDA. Other adverse event reporting systems such as the Yellow Card scheme in the United Kingdom, and the Canada Vigilance Program in the Canadian jurisdiction that collect and assess spontaneous reports of adverse drug reactions from patients and health professionals are also viable options for expanding the data [25].

### Limitations

The study findings should be interpreted cautiously with the following caveats. Limited numbers of drugs were assessed in the study given the finite number of FDA-CDER-approved orphan drugs. Uncertainty remains regarding the association between postmarketing safety events and other approval factors that yielded insignificant findings in the current analysis. Underestimating long-term PMSE is likely, given that the reported safety events of orphan drugs are often based on a small population. This, along with the inherent differences between rare disease and orphan drug definitions employed by various drug regulatory authorities, could discount the generalisability of our findings. Furthermore, since the estimation of safety events is based on reports from the drug surveillance system, no comparison between placebo and intervention arms could be made and interpreting the results of our study should be taken cautiously.

It should also be noted that the study only examined newly approved chemical or biological agents with an orphan drug designation. Drugs initially approved for common disease conditions and later repurposed as orphan drugs were not considered. At the same time, drugs with orphan drug designation could be extended to indications of common diseases when adequate and high-quality clinical evidence becomes available, highlighting the importance of safety surveillance when drugs are used on a broader population. For drug developers and regulators there is an inherent trade-off between the demand for life-saving drugs with early treatment access and the need to gather conclusive evidence about the real-world effectiveness and long-term safety. As such, additional safety information discovered after a drug has been approved is both expected and appropriate.

## Conclusions

Frequent postmarketing safety-labelling updates occur among FDA-approved drugs with orphan drug designation and expedites approval, particularly for drugs approved for long-term use or approved with boxed warning. Labelling changes related to severe safety events are uncommon and focus mainly on the reinforcement of initial boxed warning. Drug regulatory systems, collectively with research partners and sponsors, must strive to maintain timely medication safety surveillance and obtain more evidence to better inform clinicians and stakeholders about the risks and benefits of orphan drugs.

## Supplementary Information

**Additional file 1**. Data extraction flowchart

**Additional file 2**. Extracted data list and data source

**Additional file 3**. Orphan drugs with newly added boxed warning

**Additional file 4**. Proportion of orphan drugs affected by severe postmarketing safety events

**Additional file 5**. Schoenfeld residuals plots for proportional hazard assumption checking

## Data Availability

The datasets used and analysed during the current study are available from the corresponding author on reasonable request.

## Abbreviations

FDA: Food and Drug Administration
PMSEs: Postmarketing Safety Events
SPSEs: Severe Postmarketing Safety Events
IRR: Incidence Rate Ratio
95% CI: 95% Confidence Interval
NME: New Molecular Entity Application
BLA: Biologics License Application
EMA: European Medicines Agency
PDFUA: Prescription Drug User Fee Act
S.D.: Standard Deviation
IQR: Interquartile Range
