# Supplementary figures and images for: Postmarketing safety of orphan drugs: a longitudinal analysis of the US Food and Drug Administration database between 1999 and 2018
Source: Orphanet J Rare Dis. 2022 Jan 4;17:3. doi: 10.1186/s13023-021-02166-9 (PMC8728968; doi:10.1186/s13023-021-02166-9)

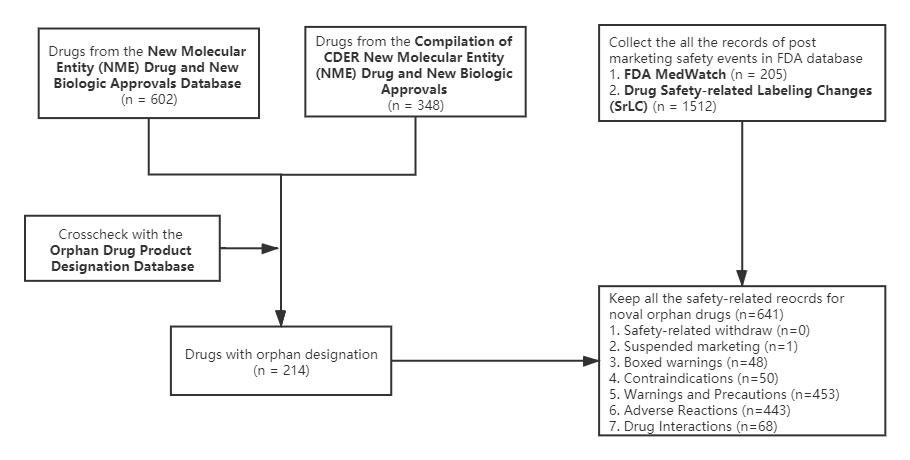

Supplement: Supplementary file 1 — Additional file 1. Data extraction flowchart [file 13023_2021_2166_MOESM1_ESM.png]

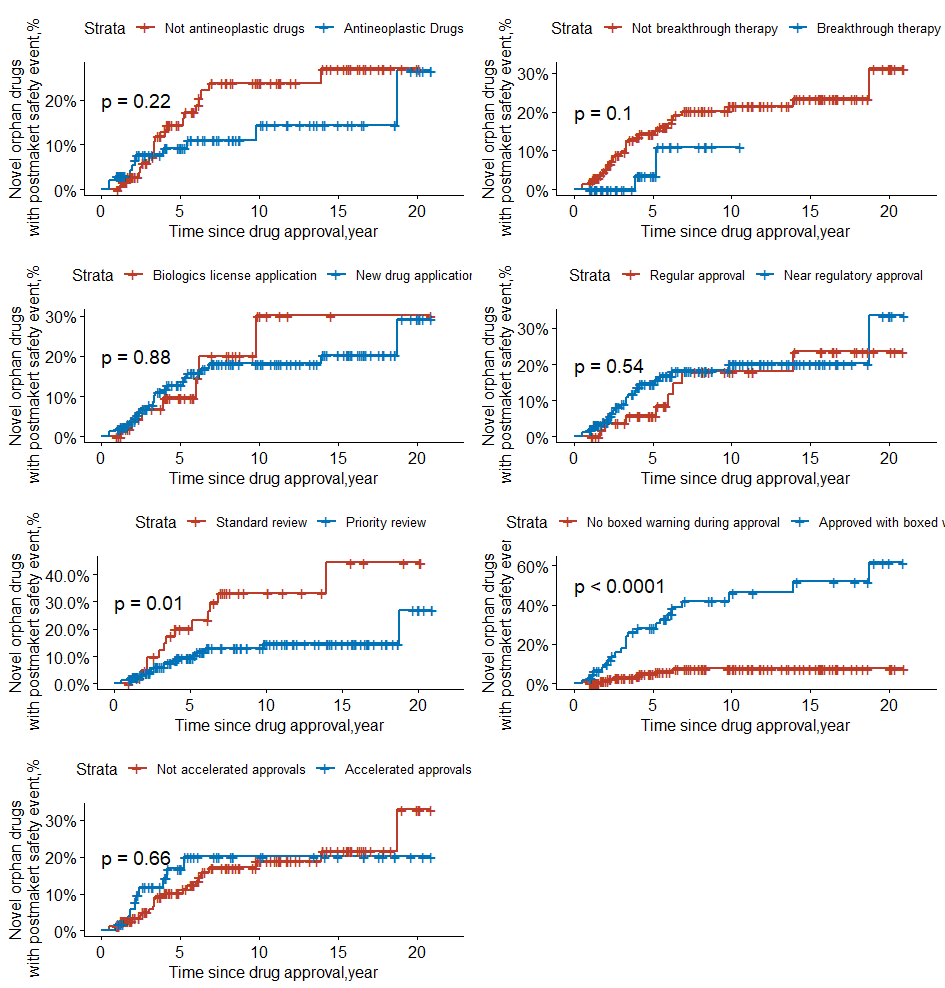

Supplement: Supplementary file 4 — Additional file 4. Proportion of orphan drugs affected by severe postmarketing safety events [file 13023_2021_2166_MOESM4_ESM.tiff]

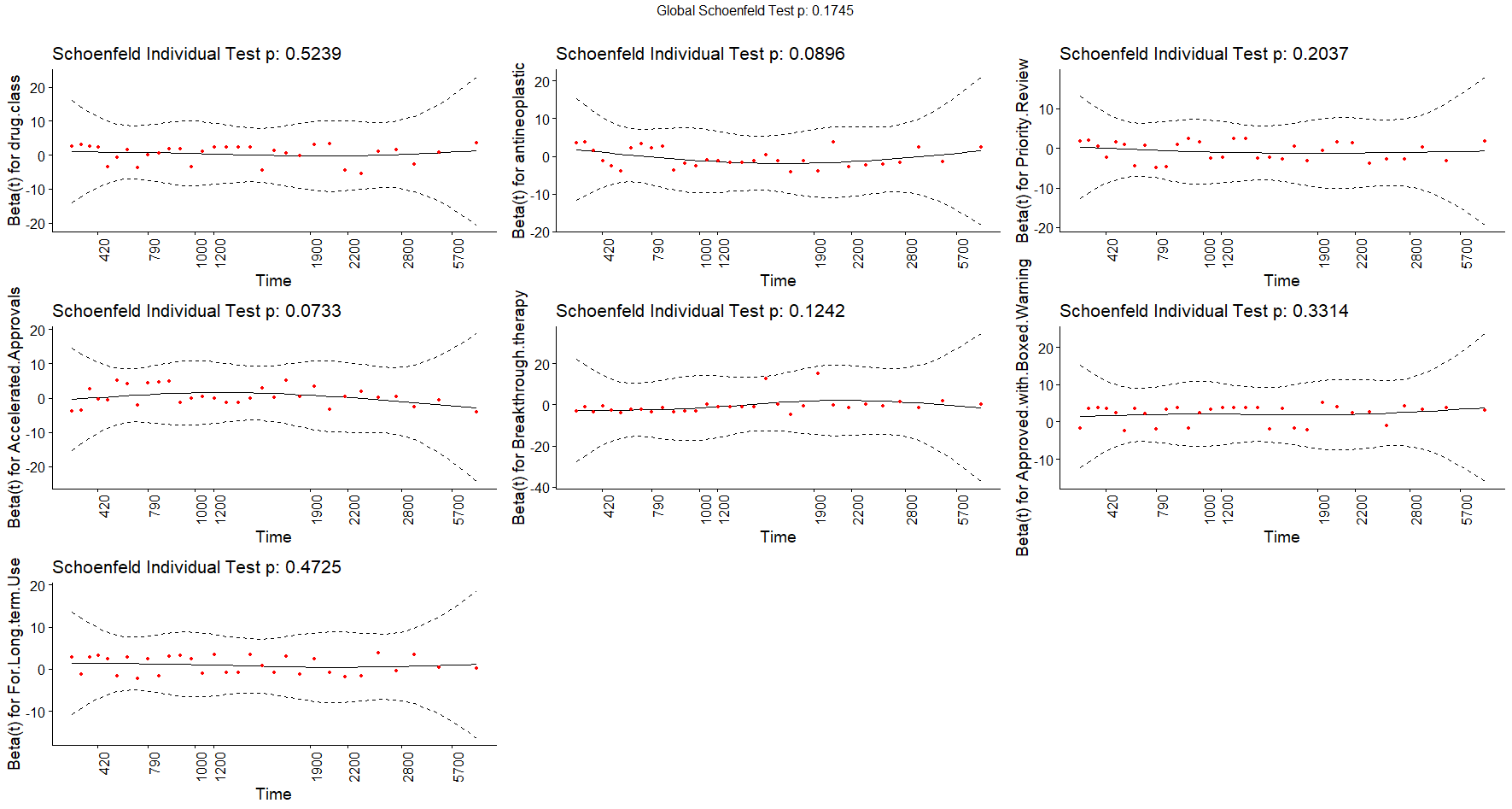

Supplement: Supplementary file 5 — Additional file 5. Schoenfeld residuals plots for proportional hazard assumption checking [file 13023_2021_2166_MOESM5_ESM.png]
