# Supplementary material for: Postmarketing safety of orphan drugs: a longitudinal analysis of the US Food and Drug Administration database between 1999 and 2018
Source: Orphanet J Rare Dis. 2022 Jan 4;17:3. doi: 10.1186/s13023-021-02166-9 (PMC8728968; doi:10.1186/s13023-021-02166-9)
Supplement: Supplementary file 2 — Additional file 2. Extracted data list and data source [file 13023_2021_2166_MOESM2_ESM.docx]

**Supplementary material 2.** Extracted data list and data source

|  | **Column** | **Description** | **Website/Remark** |
| --- | --- | --- | --- |
| 1 | Application Number / Biologics License Application (BLA) Number | The drug identifier for crosscheck in the Drugs@FDA, NOT for analysis. | Most of the left information can be extracted from:  [New Molecular Entity (NME) Drug and New Biologic Approvals](https://www.fda.gov/drugs/nda-and-bla-approvals/new-molecular-entity-nme-drug-and-new-biologic-approvals) 2015 to 2018  and  [New Molecular Entity (NME) Drug and New Biologic Approvals Archive files](https://wayback.archive-it.org/7993/20170404174205/https:/www.fda.gov/Drugs/DevelopmentApprovalProcess/HowDrugsareDevelopedandApproved/DrugandBiologicApprovalReports/NDAandBLAApprovalReports/ucm373420.htm) 1999-2014. |
| 2 | Proprietary Name / Brand Name | The exclusive name of a drug substance or drug product owned by a **company** under trademark law regardless of registration status with the PTO. Just collect for crosscheck. |  |
| 3 | Established Name / Proper Name | The designated **FDA Official name**, the compendial name, the USAN Council name or the common or usual name, generic name, or chemical name. This is universal name which is easy to search and compare by countries. However, FDA may designate an established name in cases where a monograph does not exist. (For IBALIZUMAB, it will add UIYK after that.) |  |
| 4 | Applicant | The name of pharmaceutical companies. NOT for analysis. |  |
| 5 | Review classification for expedited programs | The review classification includes:  P - Priority Review - Significant improvement compared to marketed products, in the treatment, diagnosis, or prevention of a disease.  S - Standard Review - Products that do not qualify for priority review.  O - Orphan Designation - Pursuant to Section 526 of the Orphan Drug Act (Public Law 97-414 as amended). | Review classification can be double check with the information in the FDA page of [fast track](https://www.fda.gov/drugs/nda-and-bla-approvals/fast-track-approvals), [breakthrough therapy](https://www.fda.gov/drugs/nda-and-bla-approvals/breakthrough-therapy-approvals), and [accelerate approval](https://www.fda.gov/drugs/nda-and-bla-approvals/accelerated-approvals).  [Drug@FDA](https://www.accessdata.fda.gov/scripts/cder/daf/index.cfm) is used for cross check. |
| 6 | >=1 Expedited program |  | Generated by formula. When the drug has 1 or more of the above expedited programs, pls shows ‘Y’. |
| 7 | Therapeutic Area | Therapeutic Area of NME was classified according to ‘ATC/DDD index’. | <https://www.whocc.no/atc_ddd_index/>  For drugs that cannot be found in, AYLC and MF independently conduct the categorization. |
| 8 | For long-term use | It will be defined as ‘Y’, if there are following keywords such as “long-term”, “cancer”, “chronic”, and “repeat” in the indications.  Whether the original indication of NME was intended for long-term use, which defined as chronic or repeated intermittent use for 6 months or longer, was judged based on information in the ‘Indication and Usage’ and ‘Dosing and Administration’ sections of the initially approved label, or in information regarding length of treatment found in the ‘Clinical Studies’ section of the label if the length is not clearly stated in previous sections. |  |
| 9 | Submission date | We recorded the submission date and approval date of the original NME/BLA registration in FDA through Approval Letter and Administrative Document(s) & Correspondence obtained in ‘Drugs@FDA’ Database. | <https://www.accessdata.fda.gov/scripts/cder/daf/index.cfm> |
| 10 | Approval date | Orphan Drug designation status of NME was obtained by querying ‘Orphan Drug Product designation database’. |  |
| 11 | Approved with Boxed warning, | We recorded whether drugs was initially approved with Boxed warning according to the original insert retrieved in ‘Drugs@FDA’ Database. |  |
| 12 | Met PDUFA goal | We also retrieved data through Approval Letter and Administrative Document(s) & Correspondence found in ‘Drugs@FDA’ Database to judge whether the application of original NME/BLA met the PDUFA (Prescription Drug User Fee Act) goal date, take into account of any date extension due to major amendment in application, whether the application had such major amendment causing extension, whether the application approved near the PDUFA goal date, i.e. 60 days or less, and whether the application met its first-cycle PDUFA goal date, i.e. the original goal date according to the PDUFA. |  |
| 13 | Label changes | Drug Safety-related Labeling Changes (SrLC) | The information about label change are saved: <https://www.accessdata.fda.gov/scripts/cder/safetylabelingchanges/>  Data prior to January 2016 will to be available on the archive pages:  <http://wayback.archive-it.org/7993/20170110235327/http:/www.fda.gov/Safety/MedWatch/SafetyInformation/default.htm> |
