# Supplementary material for: Postmarketing safety of orphan drugs: a longitudinal analysis of the US Food and Drug Administration database between 1999 and 2018
Source: Orphanet J Rare Dis. 2022 Jan 4;17:3. doi: 10.1186/s13023-021-02166-9 (PMC8728968; doi:10.1186/s13023-021-02166-9)
Supplement: Supplementary file 3 — Additional file 3. Orphan drugs with newly added boxed warning [file 13023_2021_2166_MOESM3_ESM.docx]

**Supplementary material 3**. Orphan drugs with newly added boxed warning

| **Brand name** | **Generic Name** | **Expedited programs** | **For Long-term Use** | **Approved indication** | **Therapeutic Area** | **New boxed warning** |
| --- | --- | --- | --- | --- | --- | --- |
| Arzerra | Ofatumumab | PR,AA,BT | yes | Chronic lymphocytic leukemia (CLL) refractory to fludarabine and alemtuzumab | Antineoplastic and immunomodulating agents | Hepatitis B virus reactivation and progressive multifocal leukoencephalopathy |
| Exjade | Deferasirox | PR,AA | yes | Chronic iron overload due to blood transfusions (transfusional hemosiderosis) in patients 2 years of age and older. | Various | Renal failure, hepatic failure, and gastrointestinal hemorrhage |
| Extraneal | Icodextrin | Nil | yes | Chronic renal failure during continuous ambulatory peritoneal dialysis (CAPD) or automated peritoneal dialysis (APD). | Blood and Blood forming organs | Dangerous drug-device interaction found in blood glucose monitoring devices using glucose dehydrogenase pyrroloquinoline quinone (GDH PQQ-) or glucose-dye-oxidoreductase (GDO)-based methods. |
| Ocaliva | Obeticholic acid | PR,AA | no | Primary biliary cholangitis (PBC) in combination with ursodeoxycholic acid (UDCA) in adults with an inadequate response to UDCA, or as a monotherapy in adults unable to tolerate udca | Alimentary tract and metabolism | Hepatic decompensation and failure in incorrectly dosed pbc patients with child-pugh class b or c or decompensated cirrhosis |
| Onfi | Clobazam | Nil | No | Seizures associated with Lennox-Gastaut syndrome (LGS) | Nervous system | Profound sedation, respiratory depression, coma, and death may occur from concomitant use with opioids |
| Spinraza | Nusinersen | PR | Yes | Spinal muscular atrophy in pediatric and adult patients | Musculo-skeletal system | Serious infections with lumbar puncture, have been observed, as well as hydrocephalus, aseptic meningitis, and hypersensitivity reactions. |
| Xospata | Gilteritinib | PR | yes | For the treatment of adult patients who have relapsed or refractory acute myeloid leukemia (aml) with a fms-like tyrosine kinase 3 (flt3) mutation as detected by an fda- approved test | Antineoplastic and immunomodulating agents | Differentiation syndrome |
| Zydelig | Idelalisib | AA | yes | For relapsed follicular B-cell Non-Hodgkin Lymphoma and relapsed small lymphocytic lymphoma | Antineoplastic and immunomodulating agents | Fatal and serious toxicities: Hepatic, severe diarrhea, colitis, pneumonitis, infections, and intestinal perforation |
| * PR: Priority Review; BT: Breakthrough Therapy; AA: Accelerate Approval  * The suspension of Iclusig was due to the same safety warnings at first approval, not newly boxed warning. | | | | | | |
